# Interleukin-1 in the Response of Follicular Helper and Follicular Regulatory T Cells

**DOI:** 10.3389/fimmu.2019.00250

**Published:** 2019-02-27

**Authors:** Paul-Gydéon Ritvo, David Klatzmann

**Affiliations:** ^1^Sorbonne Université, INSERM, Immunology-Immunopathology-Immunotherapy (i3), Paris, France; ^2^AP-HP, Hôpital Pitié-Salpêtrière, Biotherapy (CIC-BTi) and Inflammation-Immunopathology-Biotherapy Department (i2B), Paris, France

**Keywords:** plasma cell, antibody production, immunoregulation, immunotherapy, germinal centers, Tfr cells, Tfh cells

## Abstract

The role of interleukin-1 in the regulation of humoral responses is poorly documented, in contrast to its role in inflammation. Recent findings suggest there is an interleukin-1 axis in the follicular T cell control of B cell responses, involving interleukin-1 receptors (IL-1R1 and IL-1R2) and receptor antagonists (IL-1Ra). Here, we revisit the literature on this topic and conclude that targeting the interleukin-1 pathway should be a valuable therapeutic approach in many diseases involving excessive production of (auto)antibodies, such as autoimmune diseases or allergy.

## Introduction

Interleukin-1 (IL-1) is known as the key cytokine of innate immune responses and has been described as the “quintessential inflammatory cytokine” (1). IL-1 is predominantly produced by monocytes and macrophages (2, 3) following an external stimulus such as through Toll-Like Receptor (TLR) activation. IL-1 pleiotropic functions have so far mainly been linked to inflammation, orchestrating a first line of defense against pathogens (4). IL-1 has systemic effects that trigger fever, cortisol production, and liver stimulation (with production of C-reactive and complement proteins) and local effects on innate and adaptive immune cell stimulation. The effects of IL-1 on innate immunity have been extensively studied and reviewed (4, 5). Those on adaptive immunity have been ascribed to a general amplification of T-cell responses (6) and to modulation of T cell plasticity toward Th17 cell differentiation (7, 8). Except for the known role of IL-1 in adjuvanticity (9), the involvement of IL-1 in the regulation of humoral response is poorly documented. Indeed, a thorough check of the literature, including multiple PubMed queries such as various combinations of “interleukin-1,” “IL-1,” “IL-1Ra,” “IL-1R1,” “IL-1R2,” “Tfh,” “Tfr” “follicular cells,” “humoral immunity,” “antibody production,” “autoantibody production,” and “germinal centers” did not identify relevant publications. We review here recent findings that highlight a key role of IL-1 in the regulation of follicular helper and follicular regulatory T cells, thereby controlling B cell responses.

## The IL-1 Activation Pathway

IL-1ß is part of a wide family of cytokines (IL-1α, IL-1ß, IL-18, IL-33, IL-36), receptor antagonists (IL-1Ra, IL-36Ra, IL-38) and the anti-inflammatory IL-37. The IL-1 activation pathway has been reviewed elsewhere (10). Briefly, IL-1 is produced as an inactive precursor, pro-IL-1ß, in response to pathogen-specific signals. This stimulation of innate immune cells induces the formation of the inflammasome, a molecular scaffold composed of many molecules such as NLRP3 (11). This key system activates caspase 1 (also called ICE for Interleukin-1 Converting Enzyme), an enzyme able to cleave pro-IL-1ß (12). It is worthy of note that the mechanism of IL-1ß secretion is not the conventional endoplasmic reticulum and Golgi route (13), but is not well understood yet and may depend on many parameters, such as stimulus strength and IL-1 requirement (14).

The signaling pathway following the interaction of IL-1 with its agonist receptor IL-1R1 has been described to be the same as many other signaling pathways, such as those triggered by the interaction of pathogenic components with TLR or of IL-33 with its receptor (10, 15). The first step consists of the recruitment of MyD88 to the receptors (16), and the cascade that follows—called the “canonical pathway”—which leads to the final activation of NF-kB. This ultimately activates the expression of pro-inflammatory genes such as cytokines, chemokines and adhesion molecules (17).

IL-1R2 and IL-1Ra regulate the IL-1ß / IL-1R1 interaction. IL-1R2 has an extracellular domain structurally similar to that of IL-1R1 but which lacks the intracellular domains allowing signaling. It thus acts as a decoy receptor, capturing the IL-1ß and thereby preventing IL-1R1 stimulation (18). IL-1R2 is expressed at high levels by macrophages, neutrophils and B-cells (19). IL-1Ra is a cytokine that inhibits IL-1 function by binding to IL-1R1 without producing any agonist effects, thereby preventing IL-1 binding (20, 21).

## IL-1 and the Regulation of Tfh and Tfr Cells

Antibody production by plasma cells is tightly regulated by follicular helper T (Tfh) cells. Help by Tfh cells is essential for the differentiation of B cells into antibody-producing plasma cells (22, 23). In contrast, follicular regulatory T (Tfr) cells negatively control humoral immune responses (24, 25). These cells are thought to be derived from regulatory T (Treg) cells (26, 27). Their mechanisms of action are poorly known and they are thought to act by regulating the help provided by Tfh cells to B cells. However, recent findings have shown that few Tfr cells are located within the germinal centers (GCs) of LNs, where Tfh cells and plasma cells interact. Most Tfr cells are found surrounding the GCs and are likely not in contact with Tfh cells (28).

In contrast to Treg cells from which they are derived, we observed that Tfr cells do not respond to interleukin-2 (IL-2) (29). This led us to reexamine their phenotype thoroughly. In contrast to Treg cells and to previous description of the Tfr-cell phenotype, we showed that Tfr cells do not express IL-2Ra (CD25), the essential component of the high-affinity IL-2R. This is important because most previous investigations of Tfr cell biology actually reported the biology of mixtures of Tfr and Treg cells. The stringent characterization of Tfr cells allowed us to reveal a striking distribution of IL-1 receptor expression on Tfh and Tfr cells. We observed that Tfh cells express the IL-1R1 agonist receptor while Tfr cells express both the IL-1R2 decoy receptor and the antagonist IL-1Ra. The lack of CD25 expression by Tfr cells and this distribution of IL-1 receptors have also been observed by others (30, 31).

This striking distribution of the agonist receptor on Tfh cells and of the antagonist receptors/inhibitors on Tfr cells led us to hypothesize and explore a possible IL-1 axis in the regulation of humoral responses. We observed that, *in vitro*, IL-1ß activated the production of IL-4 and IL-21 by Tfh cells. These cytokines have been shown to be crucial for the T-cell help to B cells (32). This cytokine production was suppressed by Tfr cells to the same extent as by recombinant IL-1Ra (Anakinra), indicating that the suppressive effect was likely dependent on the blocking of IL-1 by IL-1R2 on the surface of Tfr cells, or on IL-1Ra produced by Tfr cells. Eventually, we showed that, *in vivo*, IL-1ß induced proliferation of Tfh cells while Anakinra significantly reduced the proportion of Tfh cells.

Altogether, we revealed an IL-1 axis regulating the germinal center responses (29) and suggested the existence of a dual regulation of T cells in secondary lymphoid organs, one between Treg and effector T cells regulated by IL-2 outside GCs and the other between Tfh and Tfr cells regulated by IL-1 inside GCs.

## IL-1 and Regulation of the Humoral Response (Figure 1)

There are no or few experiments that have investigated a direct link between IL-1 and antibody production. However, revisiting the literature, there are actually many observations indirectly supporting the involvement of an IL-1 axis in the control of humoral immunity (summarized in Table 1). First, IL-1 administration during an immunization enhanced humoral responses and led to greater antibody production (9, 38, 39), a phenomenon referred to as the “adjuvanticity of IL-1,” which was mostly thought to act by stimulation of innate immune cells that in turn would stimulate helper T cells to help B cells better. Interestingly, this observation is itself indirectly supported by the fact that many adjuvants used for immunization, such as the widely used alum, trigger enhanced IL-1 production (34). Similarly, stimulation by pathogens, which ultimately triggers antibody production, is also a strong IL-1 enhancer (35). Altogether, it appears that there is some positive correlation between production of IL-1 during immunization and the efficacy of the resulting B cell response.

**Figure 1:**
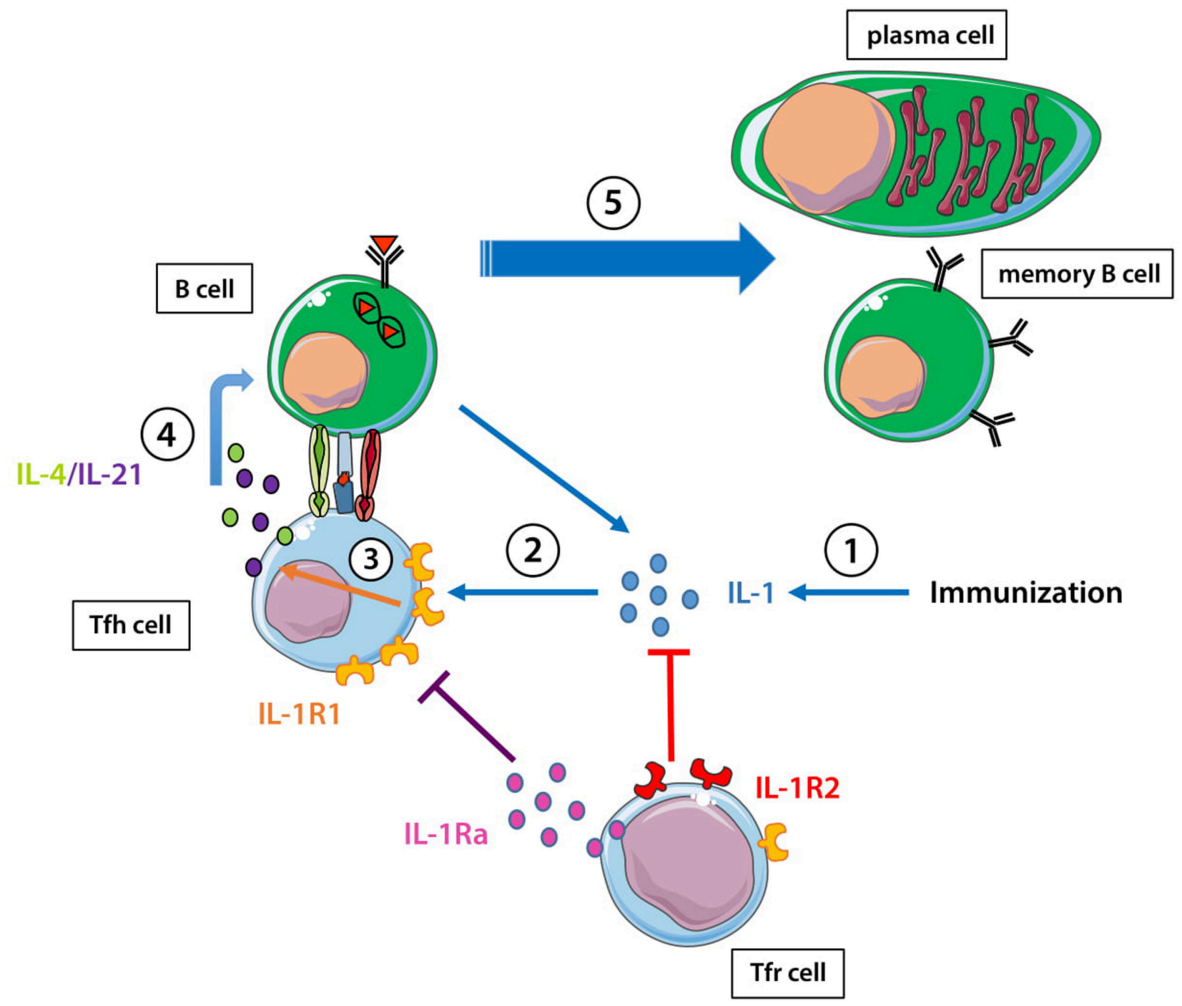
IL-1 and the regulation of the humoral response. (1) Immunization enhances IL-1 production. (2) IL-1 stimulates tfh cells through the IL-1R1, can be captured by IL-1R2 on Tfr cells, and IL-1Ra produced by Tfr cells can block the action of IL-1 on IL-1R1. (3) IL-1 stimulates IL-4/IL-21 production by Tfh cells through its binding to IL-1R1. (4, 5) IL-4/IL-21 helps B cells to become either memory B cells or antibody-producing plasma cells.

**Table 1:** Experiments supporting a correlation between IL-1 production during immunization and resulting antibody production.

**Experiment**	**Effect**	**Comment**	**References**
IL-1 as an adjuvant	Enhanced antibody response	Dose- and time-dependent adjuvanticity of interleukin 1 *in vivo*. IL-1 given 2 h after the priming dose of antigen enhanced antibody response	(9)
IL-1 as an adjuvant	Enhanced antibody response	IL-1 is an effective mucosal vaccine adjuvant when coadministered with protein immunogens, and is as effective as cholera toxin in inducing Ag-specific serum IgG	(33)
Alum induces release of IL-1	Enhanced antibody response (alum effect)	Considering the immunostimulatory activities of these cytokines and the ability of IL-1β to act as an adjuvant, the results suggest a mechanism for the adjuvanticity of alum	(34)
Cholera toxin induces release of IL-1	Enhanced antibody response	Cholera toxin (CT) is a strong systemic and mucosal adjuvant that greatly enhances IgG and IgA immune responses. It stimulates IL-1 production	(35)
IL-1α/β-deficient mice	Reduced antibody production	Primary and secondary antibody production against T-dependent antigen was significantly reduced in IL-1α/β^−/−^ mice after immunization	(36, 37)
IL-1Ra-deficient mice	Increased antibody production	Primary and secondary antibody production against T-dependent antigen was significantly increased in IL-1Ra^−/−^ mice after immunization	
IL-1 effects on CD40L	Increased CD40L expression	CD40L expression on T cells was affected in IL-1^−/−^ mice, and the reduced Ag-specific B cell response in IL-1^−/−^ mice was recovered by treatment with agonistic anti-CD40 mAb both *in vitro* and *in vivo*. IL-1 enhances T cell-dependent Ab production by augmenting CD40L	(37)

Experiments using genetically modified mice not expressing the IL-1 gene or its receptors further support the importance of an IL-1 axis in the control of humoral responses. Following appropriate stimulation, IL-1-deficient mice produced significantly reduced amounts of antibodies compared to wild-type mice (36, 37). Conversely, mice deficient in the expression of IL-1Ra, an antagonist of the IL-1R1 receptor, showed increased antibody production in the same conditions (36, 37). In these studies, the effect of IL-1 on humoral immunity enhancement was shown to act through induction of co-stimulatory molecules on T cells, such as CD40L and OX40 (37), which were later found to be highly expressed on the surface of Tfh cells (40) (Table 1).

## IL-1 and the Pathophysiology of Humoral Responses

Some evidence for the involvement of IL-1 in the humoral response can also be found by looking at diseases associated with excessive levels of antibodies and/or pathogenic antibodies.

### Autoimmune Diseases

Autoantibody production is common in autoimmune diseases and frequently contributes to their pathophysiology. Much evidence of the involvement of IL-1 in the control of autoantibody production can be found in the recent literature.

One of the interesting models for the study of this role is Myasthenia Gravis (MG), a disease caused by a pathogenic anti-acetylcholine receptor (AChR) IgG1 (41). (i) IL-1ß gene polymorphisms have been found in association with MG, suggesting a possible pathogenic role of this cytokine in the disease (42); (ii) Anakinra reduced the clinical symptoms of mice with experimental autoimmune MG (EAMG) and suppressed the pathogenic anti-AChR IgG1 (41); (iii) inhibition of proteins involved in the production of IL-1ß, such as caspase-1, can regulate the humoral response in EAMG (43). In the classification by McGonagle and McDermott (44), MG is more an autoimmune than an auto-inflammatory disease. This suggests that the consequences of blocking IL-1 in this disease should mostly be due not to the blocking of IL-1 inflammatory effects, but to the contribution of IL-1 to the regulation of antibody production.

Thyroid gland autoimmune disorders also appear informative. (i) A recent large-scale study found an association with the IL-1 RN (gene encoding the IL-1Ra) receptor antagonist variable number of tandem repeats (VNTR) polymorphism in Hashimoto Thyroiditis (HT) patients (45); (ii) increased percentages of circulating Tfh cells have been found in patients with autoimmune thyroid disorders, with a positive correlation between the percentages of circulating Tfh cells and the serum concentrations of anti-TSH receptor-Ab/thyroperoxidase-Ab/thyroglobulin-Ab (46); (iii) a study suggested that both promoter and exon polymorphisms of IL-1β gene have a significant role in the risk of developing Graves' disease (GD) (47); (iv) although no significant differences in IL-1β levels were found between serum from patients with HT or GD and normal controls. IL-1β mRNA and protein levels in peripheral blood mononuclear cells of HT patients were found to be significantly higher than those of patients with GD, which were in turn higher than the level in normal controls; (v) IL-1β mRNA was also increased in thyroid gland tissue from patients with HT compared to those with GD, and this was accompanied by increased local infiltration of monocytes into thyroid tissues; correlation analysis validated the association of high IL-1β levels with the pathogenesis of HT and led to the suggestion that IL-1β may be an active etiologic factor in the pathogenesis of HT and thus represent a new target for novel diagnostics and treatment (48).

Rheumatoid or systemic diseases could also be studied from this point of view. For instance, in rheumatoid arthritis, anti-CCP antibodies were more frequently found in the rheumatoid arthritis subgroup with high levels of cytokines, including IL-1 (49). In systemic lupus erythematosus (SLE) models, mice deficient in the IL-1ß gene were found to be resistant to induction of experimental SLE and developed lower levels of anti-dsDNA antibodies, as compared to control mice (50). Bay11-7082—a broad-spectrum inhibitor with anti-inflammatory activity against multiple targets (51)—reduced autoantibody production and renal immune complex deposition in MRL/lpr mice via inhibiting NLRP3 inflammasome and NF-κB activation (52). Compared to wild-type mice, caspase-1^−/−^ mice had significant reductions in both anti-dsDNA and anti-RNP autoantibody titers, abrogation of a type I IFN signature and were protected from both renal immune complex deposition and kidney inflammation (53). A few studies reported efficacy of IL-1 blockers in SLE patients (54–56), with documented decrease in anti-dsDNA antibody levels (54, 55).

In multiple sclerosis (MS), some antibodies may be involved in the pathophysiology of some form of the disease via demyelination (57). Among these antibodies, autoantibodies directed against lipids present in myelin (58), myelin oligodendrocyte glycoprotein (MOG) (59) or myelin basic protein (60) could be pathogenic, possibly through antibody deposition and complement activation, which are frequently found in chronic active lesions (61). Supporting this, new brain lesions were reduced in MS patients receiving rituximab, an anti-CD20 drug that depletes B cells (62). Therapeutic plasma exchange has also been used to treat the disease, with success in the MS pattern involving prominent immunoglobulin and complement (63). On the other hand, IL-1ß expression in the central nervous system and in blood has been shown to be associated with disease activity, though direct mechanisms have not been established (64). IL-1R1-deficient mice were resistant to experimental autoimmune encephalomyelitis (EAE) (65), the mouse model of MS. Furthermore, treatment with IL-1Ra has some protective effect on rat EAE as it reduces the duration and severity of the disease (66). Altogether, this could be suggestive of an effect of the IL-1 axis on the disease through limitation of pathogenic antibody production in MS.

In celiac disease, individuals develop an immune reaction to gluten, mainly composed of IgA antibodies. Polymorphism of IL-1 has been associated with susceptibility to celiac disease (67) and IL-1ß is associated with the disease, though its mechanism of action is unknown (68). Finally, we recently showed that the TCR repertoires of Tfh and Tfr cells from spleens of immunised mice were surprisingly diverse and mostly composed of mildly expanded clonotypes suggesting a major bystander activation during the immune response in the GCs (69). It remains to investigate the contribution of IL-1 to this bystander activation and its possible relation to autoantibody formation.

### Hypersensitivity and Allergy

Allergy also appears of interest in supporting the involvement of IL-1 in antibody production. In 2012, preliminary results suggested that IL-1ß was involved in the development of antigen-specific Tfh cells in the airways (70). Since these results, it has been shown that exposure to IL-1ß in conjunction with ovalbumin leads to significant increases in the levels of specific anti-OVA IgE and IgG (71, 72). Mice that are deficient in the IL-1R1 receptor and sensitized to peanut for 4 weeks showed a large decrease in serum levels of peanut-specific IgE antibodies, as well as anti-peanut IgG1 antibodies. Numbers of Tfh and GC B cells were also dramatically decreased in IL-1R1^−/−^ mice compared to wild-type mice (73). The role of IL-1 in the pathogenesis of allergies was also suggested by studies showing, for instance, that administration of IL-1Ra to pigs reduced IgE production. Finally, in humans, polymorphisms of IL-1-related genes have been associated with susceptibility to allergic rhinitis (74).

## Lessons from Therapeutic Trials

Different molecules targeting IL-1 have been or are currently being developed. Among them are monoclonal antibodies directed against IL-1 (Canakinumab, Gevokizumab for instance), a human recombinant IL-1Ra (Anakinra) or a soluble decoy receptor (Rilonacept) (75). Despite widespread use of these IL-1 inhibitors in patients with autoimmune disease, very little has been reported concerning a modification of the humoral response. The only possibly relevant findings are of an increased incidence of infection compared with placebo, but no data have been presented that could support a link with regulation of humoral responses (76).

## Conclusions–Perspectives

The notion that an IL-1 axis might control humoral immune responses by Tfh and Tfr cells is just emerging. Although it was well known that IL-1 has an important role in immune responses that lead to antibody production, this role was mostly assigned to direct stimulation of an innate immune response, which in turn would control the T and B cell response independently of IL-1. The discovery of a peculiar distribution of IL-1 receptors and IL-1 antagonists on Tfh and Tfr cells led us to revisit the role of IL-1 in the control of antibody production. It is now clear that most Tfh cells from the GCs express IL-1R1 and that in a pure *in vitro* system the addition of IL-1 directly stimulates Tfh to produce the two main B-cell activation cytokines. Furthermore, IL-1 alone expands Tfh *in vivo*. Thus, a direct role of IL-1 in the activation of Tfh cells appears important for antibody production.

The role of Tfr in controlling this regulation is less well documented. Tfr cells express IL-1R2 and IL-1Ra and are thus equipped to interfere with IL-1-mediated activation of Tfh cells. Recent work has localized most Tfr cells around and not inside GCs, which would be compatible with a role in capturing/neutralizing IL-1 before it can act on Tfh cells (27). Further studies, notably assessing mice knockout for the different receptors on specific cell populations should clarify the mechanistic aspects of the IL-1 axis in Tfr/Tfh cell control of antibody production.

Meanwhile, an existing and large body of evidence indicates that targeting the IL-1 pathway should be an important, although so far ignored, therapeutic approach to many autoimmune diseases. It could work not just by reducing inflammation, which can be fueled by the antibody response, but also by directly reducing this antibody response, thus playing both sides for greater efficacy. By reducing inflammation, it can also improve the efficacy of Tregs which suppressive ability is decreased in high inflammatory context (77–80). We believe that these results should stimulate the investigation of the regulation of IL-1 in many experimental models, from autoimmunity and inflammation to allergy. Furthermore, given the availability of many drugs targeting the IL-1 pathway, and acknowledging that our experimental models of diseases do not reflect human settings of diseases well, innovative clinical trials should play a role in further elucidation of the IL-1 pathway and its therapeutic potential.

## Author Contributions

All authors listed have made a substantial, direct and intellectual contribution to the work, and approved it for publication.

### Conflict of Interest Statement

The authors declare that the research was conducted in the absence of any commercial or financial relationships that could be construed as a potential conflict of interest.
